# Jolkinolide B induces apoptosis and inhibits tumor growth in mouse melanoma B16F10 cells by altering glycolysis

**DOI:** 10.1038/srep36114

**Published:** 2016-10-31

**Authors:** Caixia Gao, Xinyan Yan, Bo Wang, Lina Yu, Jichun Han, Defang Li, Qiusheng Zheng

**Affiliations:** 1 Binzhou Medical University, Yantai 264003, China; 2 Key Laboratory of Xinjiang Endemic Phytomedicine Resources, Pharmacy School, Shihezi University, Ministry of Education, Shihezi 832002, China; 3 Institute for Advancing Translational Medicine in Bone and Joint Diseases, School of Chinese Medicine, Hong Kong Baptist University, Hong Kong SAR 999077, China

## Abstract

Most cancer cells preferentially rely on glycolysis to produce the energy (adenosine triphosphate, ATP) for growth and proliferation. Emerging evidence demonstrates that the apoptosis in cancer cells could be closely associated with the inhibition of glycolysis. In this study, we have found that jolkinolide B (JB), a bioactive diterpenoid extracted from the root of *Euphorbia fischeriana Steud*, induced tumor cells apoptosis and decreased the production of ATP and lactic acid in mouse melanoma B16F10 cells. Furthermore, we found that JB downregulated the mRNA expression of glucose transporter genes (*Glut1, Glut3* and *Glut4*) and glycolysis-related kinase genes (*Hk2* and *Ldha*) in B16F10 cells. Moreover, treatment with JB upregulated the mRNA expression of pro-apoptosis genes (*Bax)*, downregulated the mRNA expression of anti-apoptosis genes (*Bcl-2, Caspase-3* and *Caspase-9*), decreased the potential of mitochondrial membrane and increased reactive oxygen species (ROS) levels in B16F10 cells. Finally, intragastric administration of JB suppressed tumor growth and induced tumor apoptosis in mouse xenograft model of murine melanoma B16F10 cells. Taken together, these results suggest that JB could induce apoptosis through the mitochondrial pathway and inhibit tumor growth. The inhibition of glycolysis could play a crucial role in the induction of apoptosis in JB-treated B16F10 cells.

In the incidence of malignant tumor, melanomas present a small subset, but it is the most deadly cutaneous neoplasm. It has been reported that malignant tumor is an increasingly common malignancy affecting a younger population when compared with most cancers^1^. Numerous risk factors for the development of melanoma have been identified, including family history of melanoma, genetic susceptibility, environmental factors, ultraviolet radiation and age related immune suppressions, which could influence the incidence rates^2^. Melanomas comprise of many genotypic and phenotypic subtypes that can promote a better adapt to survive in extreme environmental conditions and are among the tumors with larger genomic instability^3^. Given the complexity of the tumor cells, cutaneous melanomas are often difficult to cure although much progress has been made toward the combined application of surgical operation and chemotherapies. Therefore, developing a novel treatment or strategy to combat the disease has become the urgent matter.

Chinese herbal therapy has been regarded as a precious alternative means compared with modern medicine, and investigations on active components with anticancer potential and less side effects have opened up newer avenues^4^. In our previous work, many compounds, e.g. licochalcone A, licochalcone B, alternol and isoliquiritigenin, isolated from Chinese herbal, were found to significantly induce cancer cell differentiation and inhibit cell proliferation and/or cell apoptosis. In anti-melanomas compounds, we have demonstrated that alternol could decrease the expression levels of cyclin-dependent kinase 2 (CDK2) and proliferating cell nuclear antigen (PCNA), and activate CDK inhibitor1A (p21) to inhibit melanoma B16F0 cell proliferation^5^. Moreover, alternol increases the expression levels of tyrosinase, the tyrosinase-related proteins 1 and 2 to induce mouse melanoma B16F0 cell differentiation and inhibit cell proliferation, characterized by the enhanced melanin content and tyrosinase activity^6^. JB is a diterpenoid component isolated from the dried roots of *Euphorbia fischeriana Steud*. It has been reported that JB regulates proliferation and induces apoptosis in human leukemic U937 cell^7^. Another document evaluates the anti-metastatic role and mechanisms of JB on breast cancer MDA-MB-231 cells by the integrin/FAK and ERK pathways *in vitro*^8^.

Intriguingly, a recent study reported that the glycolysis-relevant enzymes may be a promising target to inhibit cancer cell proliferation and induce cell apoptosis in gastric cancer cells^9^. Aerobic glycolysis is the primary metabolic way utilized by most tumor cells to produce adenosine triphosphate (ATP) for growth and proliferation^10^. Tumor cells tend to produce large amounts of lactate from glucose, regulate ATP synthesis by regulating substrate uptake, as well as enzymes related to glycolysis, which enables them adapt to the nutrient microenvironment^11^. Interestingly, recent studies demonstrated that the inhibition of glycolysis could induces DNA degradation and cell apoptosis through reducing ATP production^12^,^13^,^14^. Collectively, inhibition of the glycolytic pathway may be an appropriate target for inhibiting tumor growth and/or inducing apoptosis. However, the effect of JB on melanoma cells apoptosis and the regulation of JB on glycolytic pathway are still underexplored. In this study, we have found that JB downregulated the mRNA expression of glucose transporter genes (*Glut1, Glut3* and *Glut4*) and glycolysis-related kinase genes (*Hk2* and *Ldha*), increased ROS level and decreased the potential of mitochondrial membrane, and subsequently induced tumor cells apoptosis in B16F10 cells. Moreover, our animal model of mouse melanoma indicated that intragastric administration of JB suppressed tumor growth and induced tumor cell apoptosis.

## Results

### JB inhibits growth and induces apoptosis of B16F10 cells

**Figure 1:**
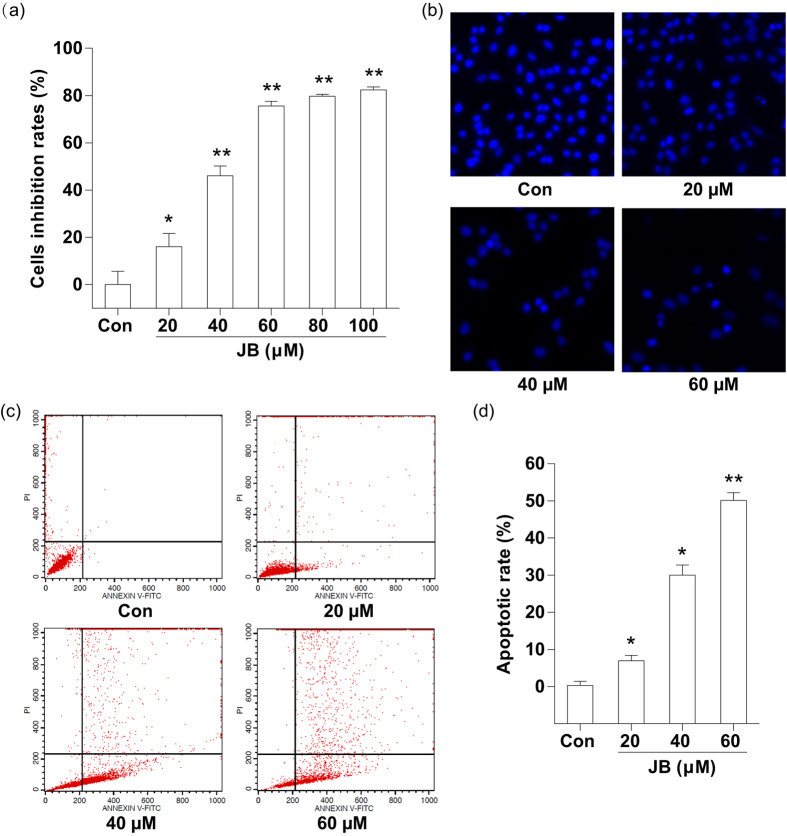
JB inhibits B16F10 cell growth and induces cell apoptosis. (**a**) Cell viability was determined by SRB assay after 24 h treatment with different concentrations of JB (20, 40, 60, 80 and 100 μM). (**b**) Morphologic measurements in B16F10 cells were carried out via Hoechst fluorescence staining. (**c**) Representative images showing the apoptotic cells after treatment with the indicated concentrations of JB. (**d**) The apoptotic rates of B16F10 cells after JB treatment. The data represent mean ± s.d. of the three independent experiments. *P < 0.05, **P < 0.01 compared with the control group.

The effect of JB on B16F10 cell proliferation was determined using SRB assay. We found a significant concentration-dependent reduction in cell viability after 24 h exposure to JB. Probit analysis showed that the median lethal concentration was 50 μM (Fig. 1a). Considering the significant proliferation inhibition on B16F10 cells induced by JB, we chose the following concentrations of JB for most of the subsequent assays: 20, 40 and 60 μM. Then, the morphological changes of B16F10 cell were examined by fluorescence microscope. The morphologic data showed notable change with the typical characteristic, e.g. cytoplasmic edema and nuclei condensed, in B16F10 cells after treatment with JB, especially 60 μM JB treatment group (Fig. 1b). Furthermore, we performed Annexin V-FITC/PI double staining to detect phosphatidyl serine (PS) externalization, which is a hallmark of early apoptosis. The apoptotic rates were markedly increased after treating with JB in B16F10 cells (Fig. 1c). The data demonstrated that the maximum concentration (60 μM) of JB induced a higher percentage (50.11 ± 2.01%) of apoptosis cells than JB-untreated group (0.27 ± 1.13%) (Fig. 1d).

### JB decreases the glycolysis of B16F10 cells through regulating the mRNA expression of glucose transporter genes and glycolysis-related genes

**Figure 2:**
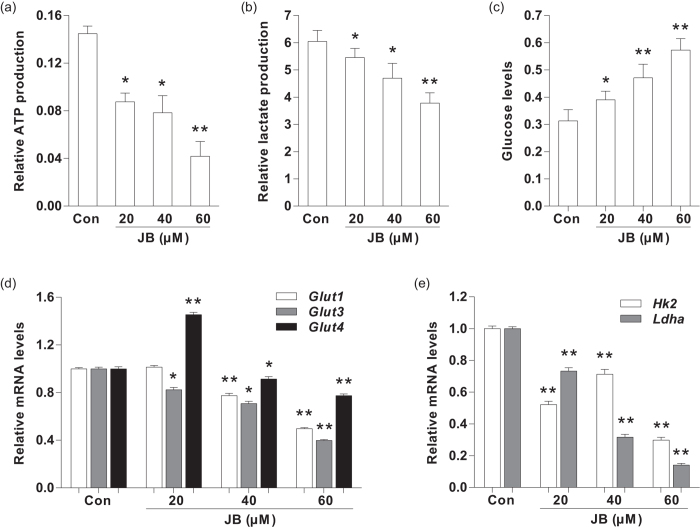
The effect of JB on glycolysis in B16F10 cells. (**a**) The intracellular ATP production were determined after treatment with JB in B16F10 cells. (**b**) Detection of lactic acid content in B16F10 cell culture medium. (**c**) The glucose levels in B16F10 cell culture medium. (**d**) The mRNA expression levels of glucose transporter genes in B16F10 cells after treatment with the indicated concentrations of JB. (**e**) The mRNA expression levels of glycolysis-related genes in B16F10 cells treated with different concentrations of JB. Note: Data are presented as mean ± s.d. from three individual treatments. *P < 0.05, **P < 0.01 compared with control group.

Considering ATP and lactic acid are the main products during glycolysis^15^, we firstly determined the ATP content in B16F10 cells and lactic acid content in culture medium to investigate whether JB could induce apoptosis through regulation of glycolysis. After 24 h incubation with JB, the levels of intracellular ATP and extracellular lactic acid were both lower in B16F10 cells after treatment with different concentrations of JB compared with JB-untreated group, especially at 60 μM (Fig. 2a,b). Correspondingly, the amount of glucose in JB-treated B16F10 cell culture medium was remarkably higher when compared to JB-untreated group (Fig. 2c). Moreover, we examined the amount of glucose in JB-treated B16F10 cell medium in the presence or absence of QVD. There is no significant difference of glucose content in cell culture medium was found between JB-treated (40 μM) B16F10 cells and JB-treated (40 μM) B16F10 cells in the presence of QVD (1 μM) (Supplementary Fig. 1a).

We next examined the mRNA expression of glucose transporters genes (*Glut1, Glut3* and *Glut4*) after 24 h treatment with JB (Supplementary Table 1). RT-PCR analysis revealed that the mRNA levels of *Glut1, Glut3* and *Glut4* were decreased in JB-treated B16F10 cells when compared to those in JB-untreated group (Fig. 2d). Subsequently, we investigated the mRNA expression of glycolysis-related kinases (*Hk2* and *Ldha*) using RT-PCR. Compared with JB-untreated group, the mRNA levels of *Hk2* and *Ldha* were both significantly lower in JB-treated B16F10 cells after JB treatment (p < 0.01) (Fig. 2e). In addition, RT-PCR analysis revealed that the mRNA levels (*Glut1, Glut3, Glut4, Hk2* and *Ldha*) didn’t change in JB-treated (40 μM) B16F10 cells when compared to these in JB-treated (40 μM) B16F10 cells in the presence of QVD (1 μM) (Supplementary Fig. 1b,c).

### JB increases apoptosis via a mitochondria-dependent pathway in B16F10 cells

**Figure 3:**
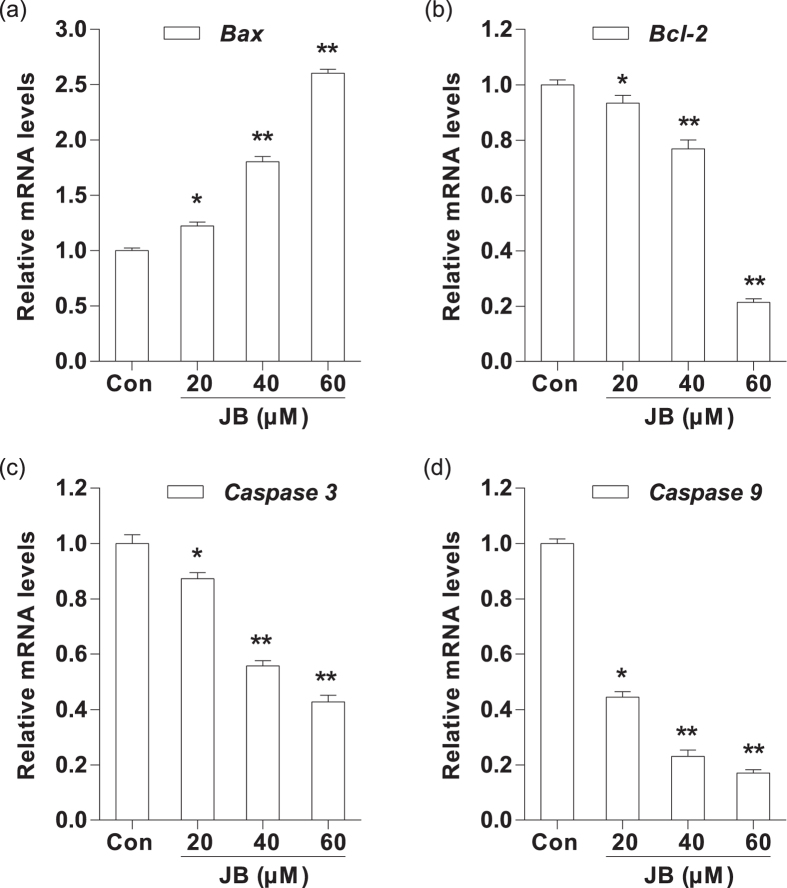
Effects of JB on the mRNA expression of apoptosis-related genes in B16F10 cells. The cells were treated with the indicated concentrations of JB for 24 h. The mRNA expression levels of *Bax* (**a**) *Bcl-2* (**b**) *Caspase-3* (**c**) and *Caspase-*9 (**d**) were analyzed by quantitative real-time PCR. Note: Data are presented as the mean ± s.d. of the three independent experiments. Mouse GAPDH mRNA was used as the internal control. All the mRNA expression levels were normalized to the mean value of the control group. *P < 0.05, **P < 0.01 compared with the control group.

To explore the molecular mechanism of JB-induced apoptosis, we evaluated the mRNA levels of apoptosis-related genes (*Bax, Bcl-2, Caspase-3* and *Caspase-9*) by RT-PCR analysis. After 24 h treatment with JB, the mRNA level of Bax was remarkably increased compared with the JB-untreated group, whereas the mRNA levels of Bcl-2, Caspase-3 and Caspase-9 were all significantly decreased after exposure to JB (*P* < 0.05). Meanwhile, we found that the above mRNA levels of apoptosis-related genes were altered in a dose-dependent manner (Fig. 3a,b). Moreover, the mRNAs expression of Bax and Bcl-2 had no significant change in JB-treated (40 μM) B16F10 cells when compared to these in JB-treated (40 μM) B16F10 cells in the presence of QVD (1 μM) (Supplementary Fig. 1d).

**Figure 4:**
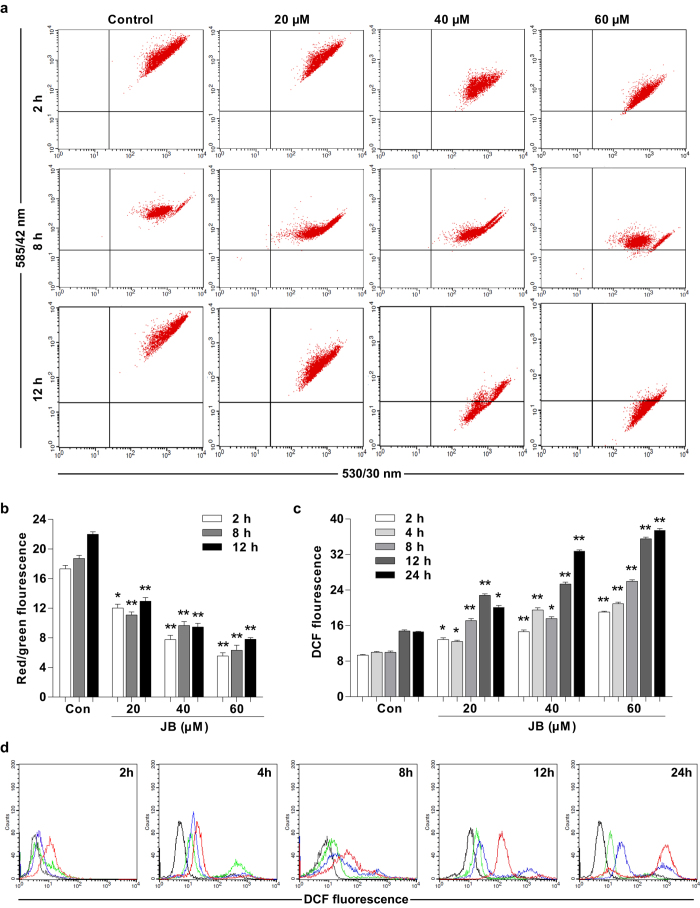
JB increases intracellular ROS production and mitochondrial damage in B16F10 cells. The cells were treated with the indicated concentrations of JB, and then mitochondrial membrane potential and intracellular ROS level were measured using Flow Cytometry. (**a**) The representative images of mitochondrial membrane potential determined by Flow Cytometry. The cells with green-positive and red-negative fluorescence were counted as depolarized cells. (**b**) The ratio of red fluorescence to green fluorescence showing mitochondrial membrane potential after JB treatment. (**c**) The fluorescent intensity of DCF showing intracellular ROS level after JB treatment. (**d**) The representative images of intracellular ROS level indicated by DCF fluorescence. Data are presented as the mean ± s.d. of the three independent experiments. *P < 0.05, **P < 0.01 compared with the control group.

To further determine whether JB-induced apoptosis could be mediated through mitochondrial dysfunction, the mitochondrial membrane potential is measured using the mitochondrion-sensitive dye JC-1. It has been reported that JC-1 either exists as a green fluorescence at lower mitochondrial membrane potentials or forms “J aggregates” with red fluorescence at higher mitochondrial membrane potentials^16^. The quantitative analysis of JC-1-stained cells revealed that a significant decrease in the ratio of red fluorescence to green fluorescence in JB-treated B16F10 cells when compared with control group (*P* < 0.05) (Fig. 4a,b). We also examined the intracellular ROS level in B16F10 cells after exposure to different concentrations of JB (20, 40 and 60 μM) for 2, 4, 8, 12 and 24 h. The intracellular ROS level in B16F10 cells was increased in a time- and dose-dependent manner (Fig. 4c,d).

### JB inhibits tumor growth, regulates the mRNA expression of glycolysis-related genes and induces tumor cell apoptosis in mouse xenograft model of murine melanoma B16F10 cells

**Figure 5:**
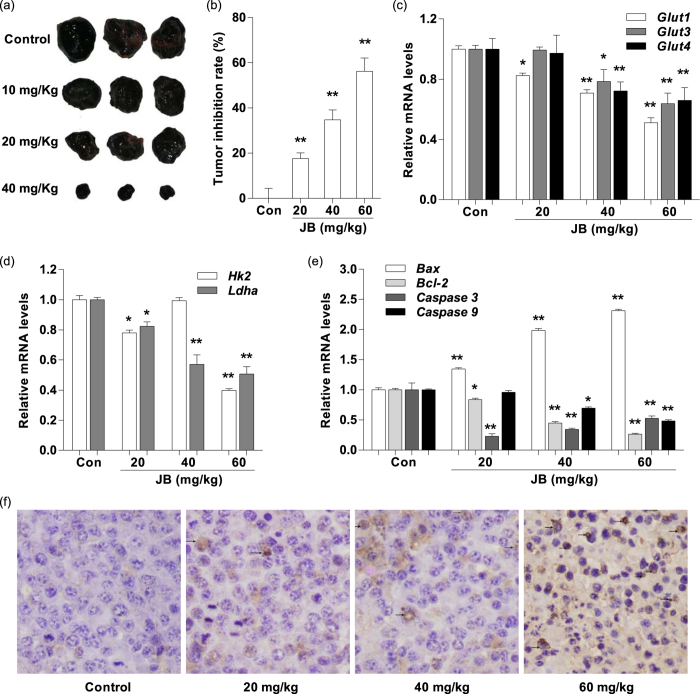
JB inhibits tumor growth, regulates the mRNA expression of glycolysis-related genes and induces tumor cell apoptosis in mouse xenograft model of murine melanoma B16F10 cells. (**a**) The representative images of the tumor morphology. (**b**) The values of tumor weight showing the inhibition effect of JB on tumor growth. (**c**) The mRNA expression levels of glucose transporter genes were examined by RT-PCR in tumor tissues. (**d**) The mRNA expression levels of glycolysis-related genes were examined by RT-PCR in tumor tissues. (**e**) The mRNA expression levels of apoptosis-related genes were examined by RT-PCR in tumor tissues. (**f**) Cell apoptosis was analyzed using TUNEL staining in tumor tissues. Data are presented as the mean ± s.d., n = 10 for each group. *P < 0.05, **P < 0.01 compared with murine melanoma xenograft mouse model without JB treatment.

Based on the encouraging findings that JB induced mouse melanoma B16F10 cells apoptosis *in vitro*, we used B16F10 tumor models to investigate whether JB could suppress tumor progression and induce tumor cell apoptosis *in vivo*. The in C57BL/6 mice bearing melanoma B16F10 cells were used as an *in vivo* model to evaluate the effects of JB. Compared with JB-untreated group (normal saline), the tumor growth rates were significantly lower in mice treated with JB at different concentrations. The tumor growth inhibition rates were 17.3%, 34.6% and 54.4% in JB-treated groups (10, 20 and 40 mg/Kg), respectively (Fig. 5a,b). To gain insight whether JB could decrease glycolysis and induce apoptosis *in vivo*, we isolated tumor tissues from the JB-treated/JB-untreated mice after sacrifice. RT-PCR analysis demonstrated that the mRNA levels of *Glut1, Glut3* and *Glut4* were down-regulated in tumor tissues from the mice after JB treatment at 20 and 40 mg/Kg (Fig. 5c). Consistent with the data from *in vitro* study, the mRNA levels of *Hk2* and *Ldha* were also significantly decreased in mice after JB treatment when compared with the JB-untreated group (*P* < 0.05) (Fig. 5d). Moreover, we examined the expression of apoptosis-related genes in tumor tissues by RT-PCR. The mRNA levels of *Bcl-2* and *Caspase9* were remarkably decreased after exposure to JB, which were consistent with the trend of the *in vitro* data (Fig. 5e). Furthermore, paraffin sections of tumor tissues were applied to the TUNEL staining. We found a gradually increased number of TUNEL-positive cells with a dose-dependent manner in tumor tissue from the JB-treated mice (Fig. 5f). Finally, there was no significant hepatotoxicity to mice after treatment with JB, as evidenced by no substantial change of serum alanine aminotransferase (ALT) and aspartate aminotransferase (AST) was found in JB-treated mice when compared to control group (Supplementary Fig. 2).

## Discussion

JB, a diterpenoid compound extracted from the dried plant roots of Euphorbia fischeriana Steud, is known to induce cell proliferation and cell apoptosis in several cancer cells^17^. However, the precise mechanisms underlying the apoptotic cell death caused by JB are mostly unclear. In the resent study, we found that JB could regulate glycolytic pathway and mitochondrial-dependent apoptotic pathway to achieve the anti-tumor effect in mouse melanoma. These results also indicated that JB-induced cell apoptosis was closely associated with inhibition of glycolysis (e.g. decreased ATP and lactic acid production) in melanoma B16F10 cells.

Previous studies have demonstrated that cell apoptosis and glycolysis are regulated through different pathways^18^,^19^, but recent evidence indicated that inhibition of glycolysis suppresses tumor cell proliferation and induce cell apoptosis. It has been reported that glycolytic enzyme hexokinase (HK) markedly inhibits the release of signal proteins from the mitochondrial intermembrane space, such as Bax and cytochrome C, through interacting with the outer membrane protein voltage dependent anion channel (VDAC). Thus, inhibition of HK expression can promote cell apoptosis and suppress cell proliferation by decreasing signal proteins release^20^. In the present experiment, our results indicated that JB could induce B16F10 cell apoptosis via decrease of the mRNA expression of HK2, one of four members of the hexokinase family.

On the other hand, lactate dehydrogenase A (LDHA) catalyzes regeneration of nicotinamide adenine dinucleotide (NAD+) from reduced nicotinamide adenine dinucleotide (NADH) and is considered as a checkpoint of glycolysis^21^. Hypoxic-induced HIF-1α protected hepatocellular mitochondrial injury and cell apoptosis through promoting LDHA expression in liver tissue^22^. In our study, we found that JB could induce B16F10 cell apoptosis though downregulating the mRNA expression of *Ldha*. Moreover, GLUTs are over-expressed in almost all cancer types and hence contribute to the increased glucose utilization that is characteristic of the glycolytic phenotype, a key signature of cancer^23^. Hypoxic-induced HIF-1α protected hepatocellular mitochondrial injury and cell apoptosis through promoting GLUT1 expression in liver tissue^22^. In our experiments, JB could block the *Glut1* expression to induce B16F10 cell apoptosis. In addition, we found that QVD, pan caspase inhibitor, had no significant effect on the glucose amount and the mRNA expression of glucose transporter gene *Glut1* and glycolysis-related genes *Hk2* and *Ldha*. Therefore, there is a link between glycolysis and apoptosis, which can be considered as one of target for JB, but its exact mechanism remains to be further confirmed.

Mitochondria have been demonstrated to play a crucial role in cell apoptosis, we therefore examined the possibility of whether mitochondrial-dependent apoptotic pathway were involved in JB-induced B16F10 cell apoptosis. The mitochondrial-dependent pathway is mainly regulated by the activity of Bcl-2 family, such as pro-apoptotic member Bax and anti-apoptotic member Bcl-2^24^. Bax increases mitochondrial membrane permeability, causing the loss of mitochondrial membrane potential and the increased of intracellular ROS, which activates the caspase cascade and initiates cell apoptosis^14^,^25^. Conversely, Bcl-2 stabilizes mitochondrial membrane permeability, thereby inhibiting apoptosis^26^. Our data indicated that JB upregulated the mRNA expression of Bax and downregulated the mRNA expression of Bcl-2, and subsequently decreased mitochondrial membrane potential and increased intracellular ROS level in melanoma B16F10 cells. Thus, JB could induce mitochondrial-mediated apoptosis through regulating Bcl-2 family members and caspases in melanoma B16F10 cells.

In conclusion, our findings reveal that decreased ATP and lactic acid production, glucose transporters and glycolytic enzymes inhibition, mitochondrial membrane potential decrease, increased intracellular ROS, upregulated anti-apoptosis genes and downregulated pro-apoptosis genes may be the mechanisms by which JB inhibits proliferation and induces apoptosis in melanoma B16F10 cells. Furthermore, intragastric injection of JB into our mouse model of melanoma suppresses tumor growth and induces apoptosis *in vivo*. Therefore, JB is a promising candidate for further development as an anti-cancer therapeutic agent.

## Materials and Methods

### Reagents

JB (molecular weight of 330.4, chemical formula C_28_H_26_O_4_, purity > 99%) was purchased from Tianjin Zhongxin Pharmaceutical Group Co., Ltd. (Tianjin, China). Culture medium (DMEM), dimethylsulfoxide (DMSO), Hoechst 33258, N-acetylcysteine (NAC), Annexin V/PI apoptosis kit, and molecular probes 2′,7′-dichlorodihy drofluorescein diacetate (H_2_DCFDA) were purchased from Sigma (St. Louis, MO, USA). JC-1 apoptosis detection kit, glucose detection kit, lactic acid detection kit, ATP detection kit were purchased from Nanjing Jiancheng Bioengineering Institute (Nanjing, China). Fetal bovine serum (FBS) was purchased from Tianjin Hao Yang Biological Manufacture Co., Ltd. (Tianjin, China). Penicillin and streptomycin were obtained from Shandong Sunrise Pharmaceutical Co., Ltd. (Shandong, China). Pan-caspase inhibitor Q-VD-OPh hydrate (QVD) was purchased from Sigma (St. Louis, MO, USA). JB was dissolved in DMSO and diluted with fresh medium to achieve the desired concentration. The final concentration of DMSO did not exceed 0.2% in the fresh medium, and DMSO at this concentration had no significant effect on the cell viability. Unless indicated otherwise, the other reagents were purchased from Sigma.

### Cell Culture

Cells were purchased from Cell Bank of the Committee on Type Culture Collection of the Chinese Academy of Sciences (Shanghai, China). The cells were maintained in DMEM medium supplemented with 10% FBS, 100 U/mL penicillin, and 100 μg/mL streptomycin at 37 °C with 5% CO_2_. The cells were split every 3 days and were diluted every day before each experiment.

### Cell Viability Assay

The cytotoxicity of the JB against B16F10 cells was tested by Sulforhodamine B (SRB) assay^27^. In brief, B16F10 cells were collected after digestion with 0.05% Trypsin-EDTA and cultured in 96-well microplates (1 × 10^5^ cells/mL, 100 μL). After 24 h culture, the cells were treated with different conventions of JB (20, 40, 60, 80, and 100 μM), respectively, for further 24 h. At the end of the incubation, the treated B16F10 cells was terminated through fixing with trichloroacetic acid (50 μL TCA, 10%) for 1 h at 4 °C. Then, the fixed cells were washed with PBS for three times and subsequently exposed to 100 μL 0.4% SRB solution (1% acetic acid) for 10 min at room temperature. This was followed by a washing with 1% acetic acid. Finally, the microplates were left to dry overnight and 150 μL Tris-HCl was added afterwards for 5 min to dissolve the protein-bound SRB on a shaker at 1500 rpm. The absorbance was measured at 540 nm using an ELISA microplate reader (Millipore Corp., Bedford, MA, USA). The data were expressed as percent cell viability compared with control group. The results reported are means of at least three separate experiments.

### Morphological observation

In order to explore whether JB induces apoptosis in B16F10 cells, the cells were planted on four-well chamber slides at 20000 cells/slide and treated with increasing concentrations of JB (20, 40 and 60 μM) for 24 h to examine apoptosis of B16F10 cells. The cells were fixed in formaldehyde with 40 g/L in phosphate buffered saline (PBS) for 20 min followed by Hoechst 33258 (10 mg/L) staining for 30 min in the dark at 37 °C. The cells in the slides were then inspected using fluorescence microscope^28^.

### Detection of Cell Apoptotic Rates by Flow Cytometry

Apoptosis was determined by staining cells with annexin V conjugated to green-fluorescent fluorescein isothiocyante (annexin V-FITC) and propidium iodide (PI) labeling^29^. Briefly, 1 × 10^5 ^cells/mL were incubated with JB for 24 h. Afterwards, the cells were washed twice with ice-cold PBS, and then 5 μL of annexin V-FITC (PharMingen, San Diego, CA, USA) and 5 μL of PI (1 mg/mL) were applied to stain cells. The status of cell staining was analyzed by using the FACStar flowcytometer (Becton Dickinson, New Jersey, USA). Viable cells were negative for both PI and annexin V-FITC; apoptotic cells were positive for annexin V-FITC and negative for PI, whereas late apoptotic dead cells displayed strong annexin V-FITC and PI labeling. Nonviable cells, which underwent necrosis, were positive for PI, but negative for annexin V-FITC.

### Detection of Intracellular Reactive Oxygen Species (ROS) Level

Fluorogenic probe 2′,7′-dichlorodihydrofluorescein diacetate (H_2_DCFDA) was used to determine the intracellular level of ROS^30^. Briefly, the cells were incubated with the indicated concentrations of JB for 2, 4, 8, 12 and 24 h. Cells were then washed in phosphate buffered saline (PBS) and incubated with 30 μM H_2_DCFDA at 37 °C for 30 min. Stained cells were washed, resuspended in PBS, and analyzed using a FACStar flow cytometer (Becton Dickinson, New Jersey, USA). Each group was acquired more than 10000 individual cells.

### Measurement of Mitochondrial Membrane Potential

In order to measure the mitochondrial membrane potential, the dual-emission potential-sensitive probe 5,5′,6,6′-tetrachloro-1,1′,3,3′-tetraethyl- imidacarbocyanine iodide (JC-1) was used. JC-1 is a green-fluorescent monomer at low membrane potential, with the membrane potential of energized mitochondria promoting the formation of red-fluorescent J-aggregates. The ratio of red to green fluorescence of JC-1depends only on the membrane potential, with a decrease being indicative of membrane depolarization^31^. B16F10 cells treated with JB of 20, 40 or 60 μM for 2, 8 and 12 h, then, the cells were loaded with 2 mg/L of JC-1 at 37 °C for 20 min. Finally, cells labeled with JC-1 can be analyzed by FACStar flowcytometer (Becton Dickinson, New Jersey, USA) using 488 nm excitation with 530/30 nm and 585/42 nm bandpass emission filters.

### Determination of glucose and lactate levels

The B16F10 cells were seeded in 6-well plates (0.8 × 10^5 ^cells/mL) then incubated with JB (20, 40 and 60 μM) in the presence or absence of QVD for 24 h. Then, remove the 6-well plate, aspirate supernatants, centrifuged at 3000 rpm for 5 minutes. Follow Nanjing Jiancheng Bioengineering Institute’s instructions, measured glucose and lactate absorbance values with the multi-plate reader at 550 nm or 530 nm, respectively.

### Determination of ATP content

The B16F10 cells were seeded in 50 ml cell culture flasks (1 × 10^5 ^cells/mL) then incubated with JB (20, 40 and 60 μM) in the presence or absence of QVD for 24 h. PBS washed cells 2 to 3 times, the collected cells were added to 300 μL hot distilled water, placed in a hot water bath (90~100 °C) homogenized broken, and heated in a boiling water bath for 10 min, mixed extract for 1 min. Then, measured ATP absorbance values with the multi-plate reader at 636 nm following the kit’s instructions.

### Quantitative real time polymerase chain reaction

Total RNA was extracted from B16F10 cells with a commercial kit (Sangon Co., Shanghai, China). RNA quality was tested using the A_260_/A_280_ ratio and cDNA synthesis was performed using Moloney murine leukemia virus reverse transcriptase with a first strand cDNA synthesis kit (Fermentas, Vilnius, Lithuania). The cDNA synthesis system was performed according to the manufacturer’s instructions. The synthesized cDNA was amplified by Olig (dT) 18 according to the instructions of a PCR amplification kit (Fermentas, Vilnius, Lithuania). The PCR primers (synthesized by Sangon Co.) and their cycling conditions were set as indicated. The reaction conditions were established by 12.5 μL 2× Quanti Fast SYBR (Sangong Co., Shanghai, China), 3 μL cDNA template, and 0.5 μL of each primer^32^.

### Animal preparation

C57BL/6 mice, aged 8–10 weeks and weighted from 18 to 22 g, were obtained from the Medical Laboratory Animal Center (SDXK (Xin) 2015-005) (Xinjiang Medical University, Xinjiang, China). The mice were maintained under standard animal care conditions (22 ± 3 °C and 60% humidity) for 7 days, with a commercial standard mouse cube diet (Shihezi University Laboratory Animal Center, Xinjiang, China) and water *ad libitum*. All animal procedures were performed in accordance with the relevant guidelines, and were approved by the Institutional Animal Care and Use Committee of Shihezi University.

### *In vivo* anti-tumor activity

B16F10 cells (2 × 10^6 ^cells/mL, 100 μL) were injected subcutaneously into the right flank of C57BL/6 mice. Tumor formation in C57 mice was monitored. Subcutaneous tumors induced by B16F10 cells in C57 mice were randomly divided into four treatment groups (10 of each group). One week after inoculation, the mice were given of 10, 20 and 40 mg/kg of JB by intragastric administration (i.g.) every day, respectively. Control mice were given the same volume of normal saline. The mice will be observed for body weight changes every two days. Seven days later after the first treatment with JB, the mice in each group will be anesthetized with 3% sodium pentobarbital via intraperitoneal injection. The blood will be collected by cardiac puncture and then centrifuged at 3000 rpm for 20 min at 4 °C to obtain the serum. The levels of serum alanine aminotransferase (ALT) and aspartate aminotransferase (AST) were determined by ELISA assay. Meanwhile, the implanted melanomas were separated and weighed, then the tumor inhibition rate (TIR) was calculated according to the following formulate: TIR (%) = (WC−WE)/WE × 100%. WC: Mean tumor weight in control group; WE: Mean tumor weight in tested groups respectively; More than 30% was regarded as having inhibitory effect^33^. During the whole experiment, conditions of the mice will be monitored. Mice with signs of severe distress or pain will be euthanized before the end of the study.

### TUNEL assay

It was carried out according to the manufacturer’s instructions. After deparaffinization and rehydration, the sections were treated with 10 mM protease K for 15 min. The slides were immersed in TUNEL reaction mixture for 60 min at 37 °C in a humidified atmosphere in the dark. A converter peroxidase (POD) was used to incubate the slides for 30 min. The slides were then analyzed using an optical microscope. To evaluate the apoptosis index of the TUNEL-stained heart tissues, we captured 10 random fields per tissue section at 400 × magnification. TUNEL index (%) is calculated as the ratio of the number of TUNEL-positive cells divided by the total number of cells^34^.

### Statistical Analysis

The data were presented as means ± s.d. from at least three independent experiments and evaluated through the analysis of variance (ANOVA) followed by student’s t-test. The values of *p < 0.05, **p < 0.01 were considered statistically significant. The analyses were performed by using the Origin 8.0 software (Origin Lab Corporation, Northampton, MA, USA).

## Additional Information

**Publisher's note**: Springer Nature remains neutral with regard to jurisdictional claims in published maps and institutional affiliations.

**How to cite this article**: Gao, C. *et al*. Jolkinolide B induces apoptosis and inhibits tumor growth in mouse melanoma B16F10 cells by altering glycolysis. *Sci. Rep.* **6**, 36114; doi: 10.1038/srep36114 (2016).

## Supplementary Material

Supplementary Information
