# Supplementary material for: Jolkinolide B induces apoptosis and inhibits tumor growth in mouse melanoma B16F10 cells by altering glycolysis
Source: Sci Rep. 2016 Oct 31;6:36114. doi: 10.1038/srep36114 (PMC5086858; doi:10.1038/srep36114)
Supplement: Supplementary Information [file srep36114-s1.pdf]

Supplementary Information

Jolkinolide B induces apoptosis and inhibits tumor growth in mouse melanoma B16F10 cells by altering glycolysis

Caixia Gao[1], Xinyan Yan[2], Bo Wang[2], Lina Yu[1], Jichun Han[1], Defang Li[1,3,*] and Qiusheng Zheng[1,*]

[1] Binzhou Medical University, Yantai 264003, China.

[2] Key Laboratory of Xinjiang Endemic Phytomedicine Resources, Pharmacy School, Shihezi University, Ministry of Education, Shihezi 832002, China.

[3] Institute for Advancing Translational Medicine in Bone and Joint Diseases, School of Chinese Medicine, Hong Kong Baptist University, Hong Kong SAR 999077, China.

* Correspondence and requests for materials should be addressed to Q.Z. (E-mail address: zqsyt@sohu.com) or D.L. (lidefang@163.com)

Supplementary Figure 1

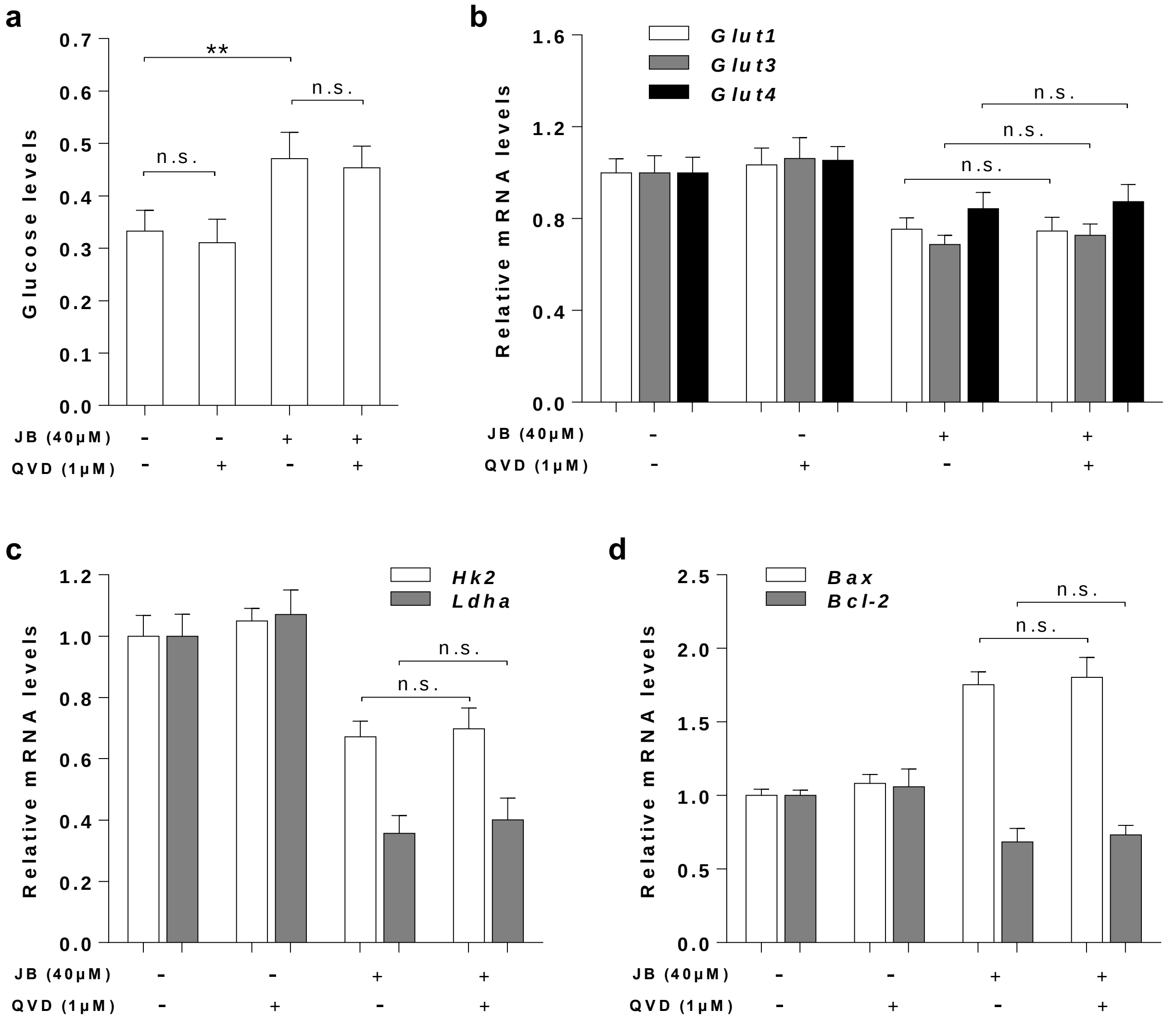

Supplementary Figure 1 Effects of QVD on the glucose amount, the mRNA expression of glycolysis-related genes and apoptosis-related genes in JB-treated B16F10 cells. The cells were treated with JB (40 μM) in the presence or absence of QVD (1 μM) for 24 h. **(a)** The glucose levels in B16F10 cell culture medium. **(b)** The mRNA expression levels of glucose transporter genes in B16F10 cells. **(c)** The mRNA expression levels of glycolysis-related genes in B16F10 cells. **(d)** The mRNA expression levels of apoptosis-related genes in B16F10 cells. **Note:** Data are presented as mean ± s.d. from three individual treatments. *P < 0.05, **P < 0.01. n.s.: no significance.

Supplementary Figure 2

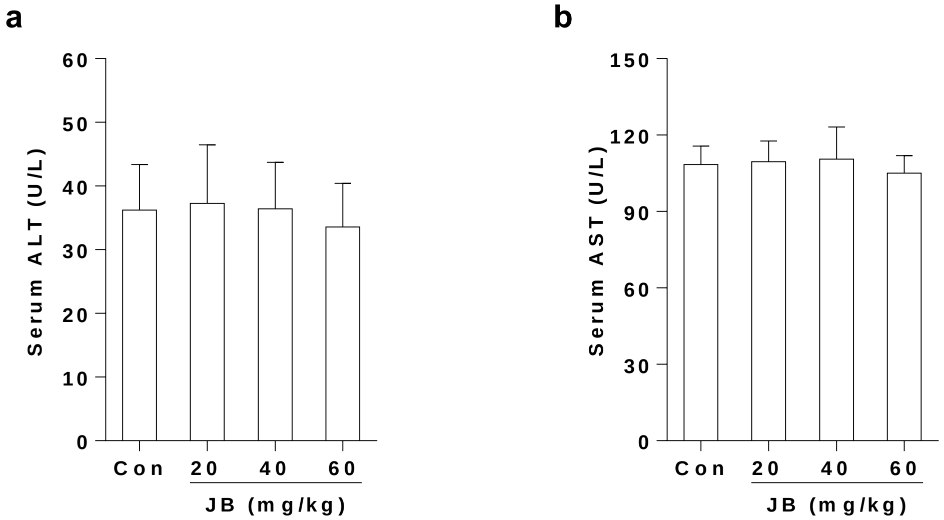

Supplementary Figure 2 The toxicity of JB in B16F10 cell tumor models *in vivo*. The serum ALT **(a)** and AST **(b)** level in tumor xenografted mice after treatment JB.

Supplementary Table 1 The primers used for quantitative real time polymerase chain reaction.

Gene	Forward Primer	Reverse primer
GAPDH	5’-ACCACAGTCCATGCCATCAC-3’	5’-TCCACCACCCTGTTGCTGTA-3’
BAX	5’-TGCTTCAGGGTTTCATCCAG-3’	5’-GGCGGCAATCATCCTCTG-3’
BCL-2	5’-GGAAATATGGCGCACGCT-3’	5’-TCACTTGTGGCCCA-3’
Caspase-3	5'-CTGGACTGTGGCATTGAGAC-3'	5'-ACAAAGCGACTGGATGAACC-3'
Caspase-9	5'-CAGTGGGCTCACTCTGAAGACC-3'	5'-ACGCGTTACTGGCATTGAGG-3'
GLUT1	5’-ATTCGGGGATTCCTGTCTCT-3’	5’-GAGTGTCCGTGTCTTCAGCA-3’
GLUT3	5’-ACCTACCAAGTGAGGGACTGC-3’	5’-ATAGAGTTGCGTCTGCCAAAG-3’
GLUT4	5’-CTTCTATTTGCCGTCCTCCTG-3’	5’-GGGTTTCACCTCCTGCTCTAA-3’
HK2	5’-CCTCAGCTGGTGAGCCATC-3’	5’-ACTGGTCAACCTTCTGCACT-3’
LDHA	5’-CTGTGTGGAGTGGTGTGAATGT-3’	5’-GCCCAGGATGTGTAACCTTTC-3’
